# Ataxia-Telangiectasia Mutated Kinase in the Control of Oxidative Stress, Mitochondria, and Autophagy in Cancer: A Maestro With a Large Orchestra

**DOI:** 10.3389/fonc.2018.00073

**Published:** 2018-03-16

**Authors:** Venturina Stagni, Claudia Cirotti, Daniela Barilà

**Affiliations:** ^1^Department of Biology, University of Rome “Tor Vergata”, Rome, Italy; ^2^Laboratory of Cell Signaling, Istituto di Ricovero e Cura a Carattere Scientifico (IRCCS) Fondazione Santa Lucia, Rome, Italy

**Keywords:** ataxia-telangiectasia mutated kinase, oxidative stress, mitophagy/autophagy, cancer, proteostasis

## Abstract

Ataxia-telangiectasia mutated kinase (ATM) plays a central role in the DNA damage response (DDR) and mutations in its gene lead to the development of a rare autosomic genetic disorder, ataxia telangiectasia (A-T) characterized by neurodegeneration, premature aging, defects in the immune response, and higher incidence of lymphoma development. The ability of ATM to control genome stability several pointed to ATM as tumor suppressor gene. Growing evidence clearly support a significant role of ATM, in addition to its master ability to control the DDR, as principle modulator of oxidative stress response and mitochondrial homeostasis, as well as in the regulation of autophagy, hypoxia, and cancer stem cell survival. Consistently, A-T is strongly characterized by aberrant oxidative stress, significant inability to remove damaged organelles such as mitochondria. These findings raise the question whether ATM may contribute to a more general hijack of signaling networks in cancer, therefore, playing a dual role in this context. Indeed, an unexpected tumorigenic role for ATM, in particular, tumor contexts has been demonstrated. Genetic inactivation of Beclin-1, an autophagy regulator, significantly reverses mitochondrial abnormalities and tumor development in ATM-null mice, independently of DDR. Furthermore, ATM sustains cancer stem cells survival by promoting the autophagic flux and ATM kinase activity is enhanced in HER2-dependent tumors. This mini-review aims to shed new light on the complexity of these new molecular circuits through which ATM may modulate cancer progression and to highlight a novel role of ATM in the control of proteostasis.

## Introduction

### Redox Homeostasis

Reactive oxygen species (ROS) are physiologically by-products of cellular metabolism and play a central role in many physiological and pathological processes including inflammation and chronic diseases such as atherosclerosis and cancer, underscoring the importance of investigating cellular pathways involved in redox homeostasis (1, 2).

Main sources of ROS are enzymes and organelles such as mitochondria (3). About 2–4% of oxygen consumed by mitochondrial oxidative phosphorylation is partially reduced and flows through membranes to activate signaling pathways that have then to be promptly turned off. Intracellular enzymatic and non-enzymatic antioxidant defense is responsible for redox homeostasis, preventing ROS accumulation (4). Together with ROS, reactive nitrogen species (RNS) are harmful molecules mostly generated by spontaneous reaction between ROS and nitric oxide signaling molecule (5).

Reactive oxygen species and RNS damage proteins as well as cellular organelles; therefore, several systems evolved to regulate and preserve a functional cellular protein pool, to ensure the quality and functionality of cellular organelles, and to finally guarantee the maintenance of proteostasis (3, 6). The autophagy-lysosomal machinery (7), the ubiquitin–proteasomal system (8), and molecular chaperones, including heat shock proteins (HSPs) (9, 10), cooperate to this aim and, indeed, they are all finely regulated by oxidative stress, which augments their functionality in order to support proteostasis and organelle quality control in challenging conditions (6).

### Ataxia-Telangiectasia Mutated Kinase (ATM) and Oxidative Stress Response

Ataxia-telangiectasia mutated kinase is a serine/threonine protein kinase, and it is a well-characterized tumor suppressor gene, which plays a central role in the nucleus in the DNA damage response (DDR). In humans, loss of function in ATM results in ataxia telangiectasia (A-T), a pleiotropic disease whose hallmarks include neurodegeneration, cancer-proneness, premature aging, radio-sensitivity, metabolic, and immune dysfunctions (11). For many years, the defect in DNA-damage response has been considered the solely responsible for A-T phenotype.

Increasing numbers of reports have described elevated readouts of oxidative stress in plasma of A-T patients, in cultured A-T fibroblasts and lymphocytes, and in tissues and cultured cells from Atm-deficient mice (12, 13). Notably, the response of A-T fibroblasts to induced oxidative stress was found defective [reviewed in Ref. (14)].

Consistently with the loss of redox homeostasis, mitochondria, are severely compromised in A-T appearing swollen and with disrupted cristae structure; as a consequence, A-T cells display mitochondrial ROS overproduction and decreased ATP levels (15). Interestingly, some of the pathological phenotypes identified in A-T, including insulin resistance, premature aging, and neurodegeneration cannot be easily connected to the well-known role of ATM in DDR, while conversely, they could be linked to the interplay between ATM and ROS (16, 17). More importantly, the administration of antioxidants to *Atm*^−/−^ mice ameliorates the disease progression and delayed cancer development (thymic lymphomas), by reducing ROS and restoring mitochondrial membrane potential (18).

These observations were at first puzzling and, more recently, they could be linked to a role of ATM in regulating cellular oxidative stress signaling. In particular, ATM is activated in the cytosol by ROS through the formation of ATM dimers *via* disulfide bonds (16, 19). Downstream to oxidative stress-dependent activation, ATM regulates a number of processes to promote restoration of redox homeostasis including adjustment of glutathione levels and activation of pentose phosphate pathway (20), regulation of mitochondrial mass, function and turnover (15, 21, 22), removal of peroxisomes *via* autophagy (23). More recently, ATM activation in response to oxidative stress has been shown to be involved in the control of proteostasis, preventing protein aggregation through a still unknown mechanism (24).

## ATM and Autophagy

The autophagy system is a finely regulated catabolic process responsible for the selective removal of cytoplasmic components (i.e., proteins, aggregates, or whole organelles) properly targeted by posttranslational modifications (ubiquitination). Basal autophagy physiologically occurs to ensure proteins turnover, maintaining intracellular homeostasis. Moreover, the autophagy system is activated by oxidative stress triggered by endogenous and exogenous stressors including nutrient starvation, hypoxia, and mitochondria and peroxisome dysfunction (25).

Ataxia-telangiectasia mutated kinase is activated in the cytosol by all the conditions listed above (16, 26); moreover, it has a role in autophagy induction (22, 27). It has been clearly demonstrated that ATM sustains autophagic pathway by inhibiting the negative regulator mTOR complex 1 (mTORC1). At the molecular level, ATM activation upon oxidative and/or nitrosative stress is responsible for the activation of LKB1/AMPK/TSC2 signaling axis, culminating with mTORC1 inhibition and relieving its repression on ULK1, which is the key protein responsible for the nucleation and formation of the autophagosome membrane, further activated by AMPK-mediated phosphorylation. This signaling pathway starting from ATM culminates in autophagy flux induction (22, 27).

The same pathway is also activated by ATM upon ROS induction under hypoxia (28). In this context, ATM promotes HIF1a stabilization by direct phosphorylation on Ser696, culminating on mTORC1 inhibition (28). Consistently, under hypoxic conditions, ATM-deficient cells fail to activate HIF1a and to inhibit mTORC1, further supporting the requirement for ATM in this pathway (28). Evidence for a role of ATM in the modulation of HIF-1a basal expression has also been provided (29, 30).

Finally, a recent work suggested that ATM regulates autophagy also by sustaining the levels and activity of ATG4C protease in cancer cells grown as mammospheres (31), characterized by low ROS levels (32). Interestingly, ATG4 proteases are the only ATG members that act as oxidative stress sensors (33). It has been demonstrated that oxidative signal leads to inactivation of ATG4s by oxidation of essential cystein residues on these proteins, at the site of autophagosome formation, thereby promoting lipidation of ATG8, an essential step in the process of autophagy (33). These data suggest that the ATM–ATG4C axis may represent a new molecular link that connects ROS, ATM, and autophagy signaling (31).

Overall, these publications suggest a role of ATM in the cytosol in regulating autophagosome formation upon exogenous and endogenous oxidative stress.

## ATM in Selective Autophagy: Mitophagy and Pexophagy

The main source of intracellular ROS are metabolically active organelles, such as mitochondria and peroxisomes (34, 35). Not surprisingly, ATM localizes to both these compartments to sense ROS increase and to activate pro-survival or pro-death intracellular pathways, depending on the intensity of the stimuli (15, 23, 36). The role of ATM in preserving mitochondrial functionality is well documented since many years. *In vivo*, loss of ATM results in mitochondria abnormalities causing ROS overproduction, strong decrease in ATP levels, and ultrastructural alterations. Moreover, the selective removal of damaged mitochondria, process known as mitophagy, is strongly impaired causing the accumulation of dysfunctional organelles (15). More recently, these evidences have been recapitulated also in neuroblastoma cells: ATM depletion results in a similar mitochondrial phenotype and mitophagy alteration, partially rescued by NAD+ cofactor replenishment (37). Taken together, these papers demonstrate the relation between ATM and mitochondria.

Although the molecular mechanism responsible for ATM function in the control of mitochondrial homeostasis deserves further investigation, it has been demonstrated that ATM activation upon mitochondrial stress or ROS increase protects cells from damage; indeed, ATM-mediated modulation of the well-characterized PINK1–Parkin pathway promotes the elimination *via* mitophagy of altered mitochondria (38).

Very recently, ATM localization to peroxisomes and its role in peroxisomes selective removal, named pexophagy, has been described. As for mitochondria, ATM localizes to peroxisomes probably to sense ROS increase and prevent damage. ATM localization in peroxisomes outer membrane is mediated by its interaction with PEX5, a peroxisome import receptor. Upon peroxisomal ROS increase, ATM-mediated PEX5 phosphorylation targets PEX5 for mono-ubiquitination and recognition by autophagic-adaptor protein (such as p62), incorporating dysfunctional organelles into autophagic vescicles (23). Very interestingly, pexophagy defects observed in ATM-deficient cells are rescued by reconstitution of ATM expression, confirming the direct role of ATM in this response (23).

The removal of damaged organelles described, so far, is also sustained by ATM-dependent induction of general autophagy, as ATM inhibits the autophagy negative regulator mTORC1, sustaining ULK1 pro-autophagic protein activation as described above (22).

Taken all together, these evidences highlight a relevant role of ATM in the cytosol: ATM ensures a prompt reply to ROS increase by activating autophagy, mitophagy, and pexophagy in order to preserve proteostasis and cellular homeostasis.

## ROS-Dependent ATM Activation and Cancer

Elevated rates of ROS have been detected in almost all cancers, where they promote many aspects of tumor development and progression (39, 40). In cancer cells, high levels of ROS can result from increased metabolic activity, mitochondrial dysfunction, peroxisome activity, increased cellular receptor signaling, oncogene activity, increased activity of oxidases, cyclooxygenases, lipoxygenases, and thymidine phosphorylase, or through crosstalk with infiltrating immune cells (41). Moreover, ROS deregulation in low oxygen tension or hypoxia condition is a common feature of all solid tumors, it is strongly associated with tumor development, malignant progression, metastatic outgrowth, and resistance to therapy and it is considered an independent prognostic indicator for poor patient prognosis in various tumor types (42). It has been largely demonstrated that ROS increase leads to proteome oxidation and instability, and alteration of the proteostasis control machine (9). More interestingly, in order to survive under stress conditions (i.e., ROS increase/hypoxia condition/starvation), many cancer cells adapt their proteostasis network and become uniquely dependent on it, an example of non-oncogene addiction (43). Individual nodes of the proteostasis network, such as Hsp90 and other HSP chaperones involved in the protein quality control networks, are currently exploited as drug targets in cancer and entered in clinical trials (44, 45).

The identification of new cytoplasmic signaling mediated by ATM in response to oxidative stress (46) and the finding that ATM can regulate networks that ensure proteins and organelles quality open the question whether these networks may contribute to A-T pathogenesis and to cancer progression (16).

It has been hypothesized for a long time that higher cancer predisposition of A-T patients depends exclusively on defects in ATM-dependent-DDR, which leads to genomic instability (11). Unexpectedly, allelic loss of the autophagy regulator Beclin-1, significantly delayed tumor development in ATM-null mice. This effect was not associated to the rescue of DNA damage signaling but rather to a significant reversal of the mitochondrial abnormalities (15). Accordingly, it has been also demonstrated that Rapamycin (mTOR inhibitor) and antioxidant treatments rescue ATM-dependent lymphomagenesis, suggesting that the dysregulation of mTORC1 and ROS contribute to A-T pathology (22). Moreover, suppression of ATM may significantly contribute to the activation of mTORC1 observed in hypoxic tumors and can promote tumor cell survival through autophagy regulation (28). Importantly, autophagy is a dichotomous phenomenon, involved in cell growth as well as in cell death, depending on its magnitude and on the cell context (47). Autophagy, as DNA damage, has been proposed to play a tumor-suppressive role in the early stages of tumorigenesis and, indeed, it is upregulated by several tumor suppressor genes; however, above a certain threshold, autophagy can also induce cell death and, if triggered appropriately, can be used as a means of killing cancer cells (48). Paradoxically, it was recently published that autophagy promotes the stem-like phenotype in breast cancer, suggesting a controversial role in cancer of autophagy (49). Interestingly, it has been reported that ATG4A and Beclin1 autophagic genes are upregulated in breast cancer stem cells (BCSCs) and are essential genes involved in BCSCs formation and maintenance (50, 51). Overall, these papers support the idea that BCSCs utilize autophagy for survival and growth, suggesting that, in this context, autophagy promotes tumor progression and tumor relapse acting as a tumor-promoting signaling. Interestingly, it was recently demonstrated that ATM kinase could have a pro-survival role in BCSCs through regulation of ATG4C gene and autophagy (31).

Finally, the expression of HSP90, a central player in the control of proteostasis, increases under stress conditions (as ROS accumulation upon oxidative stress) and, it is exploited by cancer cells to support the stability and the aberrant activity of oncoproteins overexpressed or mutated in malignancy including HER2, BCR-ABL, and EGFR (45, 52). According to this observation, HSP90 is one of the most actively pursued cancer drug targets and several different HSP90 inhibitors entered in clinical trials so far (52). Growing evidences support the idea that ATM could regulate HSP90 activity. ATM kinase can directly phosphorylate HSP90 (53, 54) although the significance of these posttranslational modifications is still largely unknown. More interestingly, we recently demonstrated that ATM activity sustains HSP90 interaction with its client protein HER2, promoting its stabilization and, therefore, sustaining HER2-dependent tumorigenicity (55, 56). These data suggest a new connection between ATM kinase and HSP90 chaperone: ATM may contribute to the control of protein quality and stability and could also modulate tumor progression *via* the regulation of this heat shock protein.

## Conclusion

**Figure 1:**
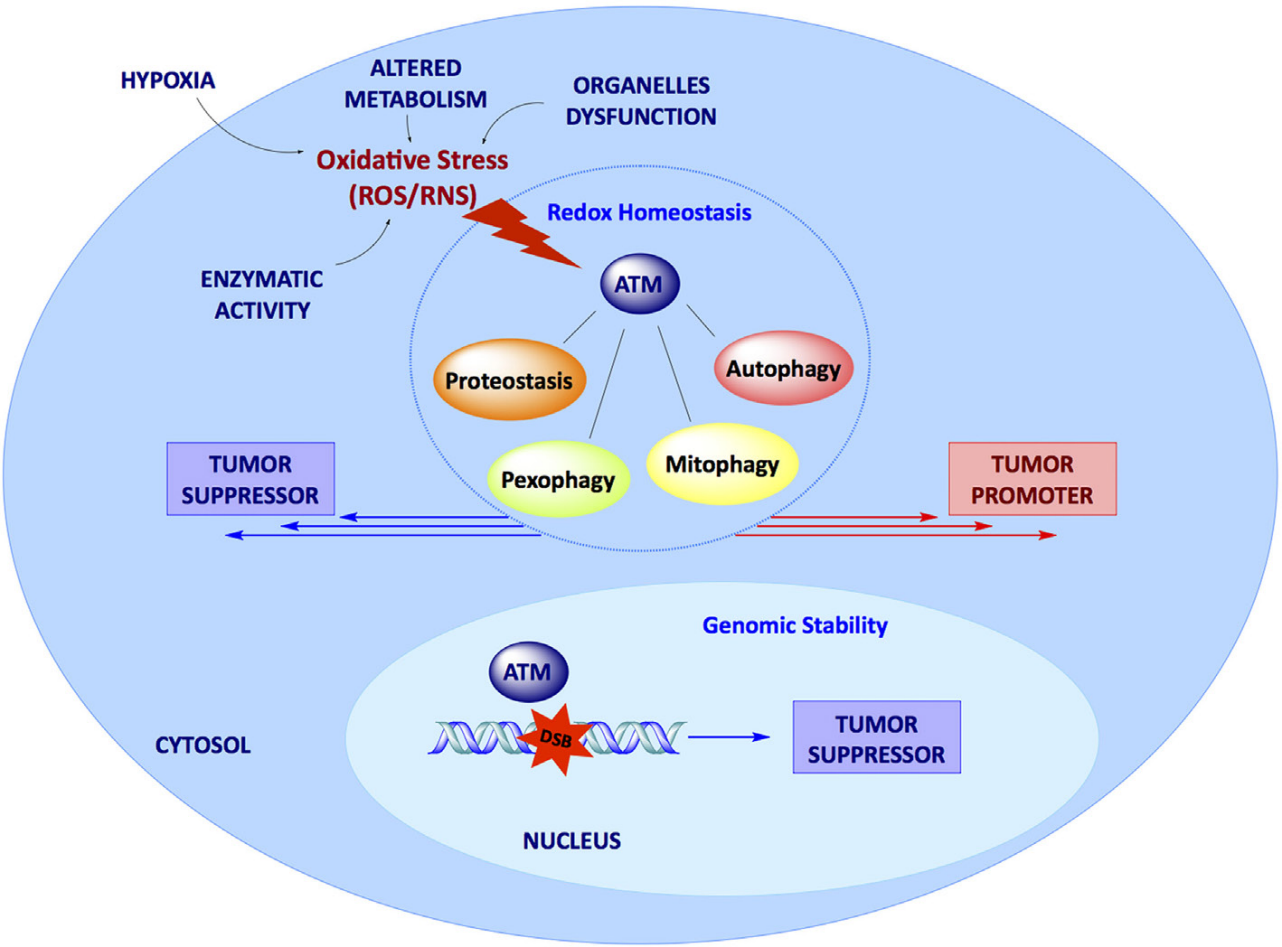
Dual role of ataxia-telangiectasia mutated kinase (ATM) in cancer. In the nucleus, DBSs activate ATM kinase, which ensures genomic stability, acting as a tumor suppressor factor. In the cytosol, ATM acts as a stress sensor, being activated upon oxidative stress to maintain intracellular redox homeostasis. Here, ATM is responsible for protein quality control and regulates several pathways such as autophagy and organelles selective removal (mitophagy and pexophagy). All these pathways may promote or prevent tumor growth depending on the specific context; the molecular mechanisms underlying the dual function of ATM still deserve further elucidation.

In conclusion, although the canonical role of ATM in the management of DNA damage defines ATM as a tumor suppressor gene, the identification of several novel functions of ATM, mostly related to its activation in response to oxidative stress and to its ability to modulate the cellular response to this insult, support multiple roles of ATM in cancer (Figure 1). ATM-dependent regulation of autophagy, mitophagy, pexophagy, and proteostasis suggest the idea that the effect of ATM expression and activity in cancer may be the result of its multiple functions in several signaling pathways and may, therefore, be strictly dependent on the specific cellular context. More studies are urgently needed to ascertain the molecular mechanisms through which this panel of cytosolic functions of ATM could modulate cancer development and therapy.

## Author Contributions

VS and DB came up with the topic for this mini-review; VS, DB, and CC wrote and edited the text.

## Conflict of Interest Statement

The authors declare that the research was conducted in the absence of any commercial or financial relationships that could be construed as a potential conflict of interest. The reviewer AM-M and the handling editor declared their shared affiliation.
